# Evaluation of changes in intestinal microbiota in Crohn’s disease patients after anti-TNF alpha treatment

**DOI:** 10.1038/s41598-021-88823-2

**Published:** 2021-05-11

**Authors:** Laura Sanchis-Artero, Juan Francisco Martínez-Blanch, Sergio Manresa-Vera, Ernesto Cortés-Castell, Marina Valls-Gandia, Marisa Iborra, Jose Maria Paredes-Arquiola, Maia Boscá-Watts, Jose Maria Huguet, Rafael Gil-Borrás, Josefa Rodríguez-Morales, Xavier Cortés-Rizo

**Affiliations:** 1 Inflammatory Bowel Disease Unit, Department of Digestive Diseases, Sagunto Hospital, Av. Ramón y Cajal S/N, 46520 Sagunto, Valencia Spain; 2 ADM-Lifesequencing S.L., University of Valencia Science Park, Carrer del Catedràtic Agustín Escardino Benlloch 9, Edificio 2, 46980 Paterna, Valencia Spain; 3 Department of preventive medicine, public health, food sciencs, toxicology and forensic medicine, Universitat de Valencia, Valencia, Spain; 4 Department of Pharmacology, Pediatrics and Organic Chemistry, Miguel Hernández University, Carretera de Valencia-Alicante S/N, 03550 San Juan de Alicante, Alicante Spain; 5 Inflammatory Bowel Disease Unit, Department of Digestive Diseases, Hospital General de Castellón, Castellón de la Plana, Spain; 6 Inflammatory Bowel Disease Unit, Department of Digestive Diseases, Hospital Universitario y Politécnico La Fe de Valencia, Valencia, Spain; 7 Inflammatory Bowel Disease Unit, Department of Digestive Diseases, Hospital Doctor Peset de Valencia, Valencia, Spain; 8 Inflammatory Bowel Disease Unit, Department of Digestive Diseases, Hospital Clínico Universitario de Valencia, Valencia, Spain; 9 Inflammatory Bowel Disease Unit, Department of Digestive Diseases, Hospital General de Valencia, Valencia, Spain; 10 Inflammatory Bowel Disease Unit, Department of Digestive Diseases, Hospital Lluís Alcanyís de Xàtiva, Valencia, Spain; 11 Department of Medicine, Universidad Cardenal Herrera-CEU, CEU Universities, Valencia, Spain

**Keywords:** Biotechnology, Microbiology, Gastroenterology, Diseases, Gastrointestinal diseases

## Abstract

Intestinal dysbiosis is key in the onset and development of Crohn’s disease (CD). We evaluated the microbiota changes in CD patients before and after a six-month anti-TNF treatment, comparing these changes with the microbiota of healthy subjects. This prospective multicenter observational study involved 27 CD patients initiating anti-TNF treatment and 16 healthy individuals. Inflammatory activity was determined at baseline, 3 and 6 months, classifying patients into responders and non-responders. Fecal microbiota was analyzed by massive genomic sequencing thought 16S rRNA amplicon sequencing before and after six months of anti-TNF treatment. The CD cohort showed a decrease in genera of the class Clostridia, short-chain fatty acid producers, and an increase in the phylum Proteobacteria (*p* < *0.01*) versus the healthy cohort. After anti-TNF treatment, the phylum Proteobacteria also increased in non-responders versus responders (13/27) (*p* < *0.005*), with the class Clostridia increasing. In addition, alpha diversity increased in responders versus non-responders (*p* < *0.01*), tending towards eubiosis. An association was found (*p* < *0.001*) in the F.prausnitzii/E.coli ratio between responders and non-responders. The F/E ratio was the most accurate biomarker of anti-TNF response (area under the curve 0.87). Thus, anti-TNF treatment allows partial restoration of intestinal microbiota in responders and the F.prausnitzii/E.coli ratio can provide a reliable indicator of response to anti-TNF in CD.

## Introduction

The microbiome consists of a highly complex structure involving thousands of microorganisms belonging to very different taxonomic classifications and consequently millions of relationships between them, making its study a great challenge^1^. As a result of advances in massive sequencing technologies, information on the bacterial genome can be obtained quickly and efficiently through the sequencing of regions of the prokaryotic 16S ribosomal RNA gene subunit^2^.

The role of intestinal bacterial microbiota has been described as key in the pathophysiology of Crohn’s Disease (CD). Studies report a decrease in biodiversity and the number of phyla Bacteroidetes and Firmicutes, such as *Faecalibacterium prausnitzii* (short-chain fatty acid (SCFA)-producing bacteria), as well as an increase in Proteobacteria phyla such as *Escherichia coli* species, characteristic of patients with this disease compared to healthy individuals^3–5^.

Anti-TNF therapy is nowadays one of the therapeutic pillars in the management of CD, but this treatment can only treat the consequences of the disease, not its possible cause. Approximately one quarter of patients will be primary non-responders to anti-TNF agents and one third of responders will experience loss of response over the years^6^. To improve the efficacy of these drugs it is essential to study why these patients do not have an optimal response. It has been described that anti-TNF treatment implies greater susceptibility to bacterial infection, caused by a loss of response capacity of the immune system^7–10^.

Monitoring inflammatory activity and prognosis in patients with CD has traditionally been limited to the control of clinical symptoms together with laboratory and imaging techniques, though not without numerous limitations and drawbacks. In this regard, several studies have shown that the greater abundance of SCFA-producing species can predict the effectiveness of infliximab (anti-TNF drug)^11,12^, while other studies have associated this greater abundance of SCFA with a sustained response to infliximab^13^ and a decrease in abundance of *Escherichia coli* after treatment with this drug^14^. Finally, one study has identified certain specific microbial profiles that correlate with disease recurrence after achieving remission with infliximab^6^.

Therefore, in this study we hypothesized that changes in the composition of the intestinal microbiota in CD patients who respond to anti-TNF drugs tend towards a microbiota similar to healthy controls versus non-responders. We also establish the relationship between *Faecalibacterium prausnitzii/Escherichia coli* as a biomarker of response to this treatment^15–17^.

## Results

### Intestinal microbiome in patients with Crohn’s Disease

**Figure 1:**
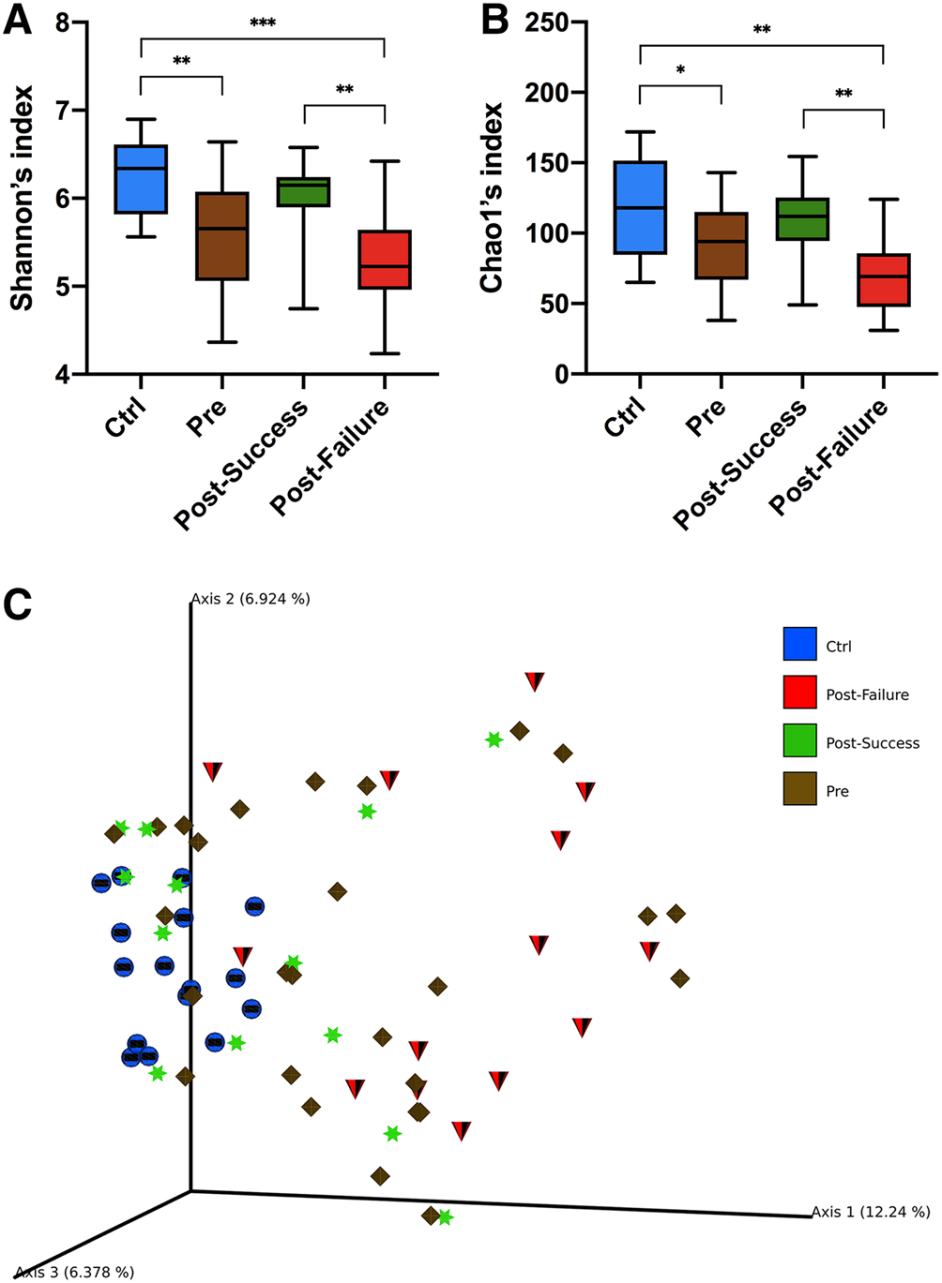
Alpha and beta diversity plots. (**A**) Box-plot corresponding to the Shannon index (alpha diversity). (**B**) Box-plot corresponding to the Chao1 index (alpha diversity). (**C**) Principal coordinates analysis corresponding to the Bray–Curtis dissimilarity index (beta diversity). Statistical analysis using the Kruskal–Wallis test with Benjamini–Hochberg correction for multiple comparisons (false discovery rate). Asterisks refer to the corrected p-value, where *p-value < 0.05, **p-value < 0.01 and ***p-value < 0.001.

Of the 43 subjects included in the study, 27 were CD patients and 16 were healthy controls. Based on the data obtained concerning alpha diversity (Shannon and Chao1 index) and beta diversity (Bray–Curtis index), a statistically significant decrease was found in the richness and diversity of microbial communities in CD patients compared to the healthy controls (Fig. 1A–C).

**Figure 2:**
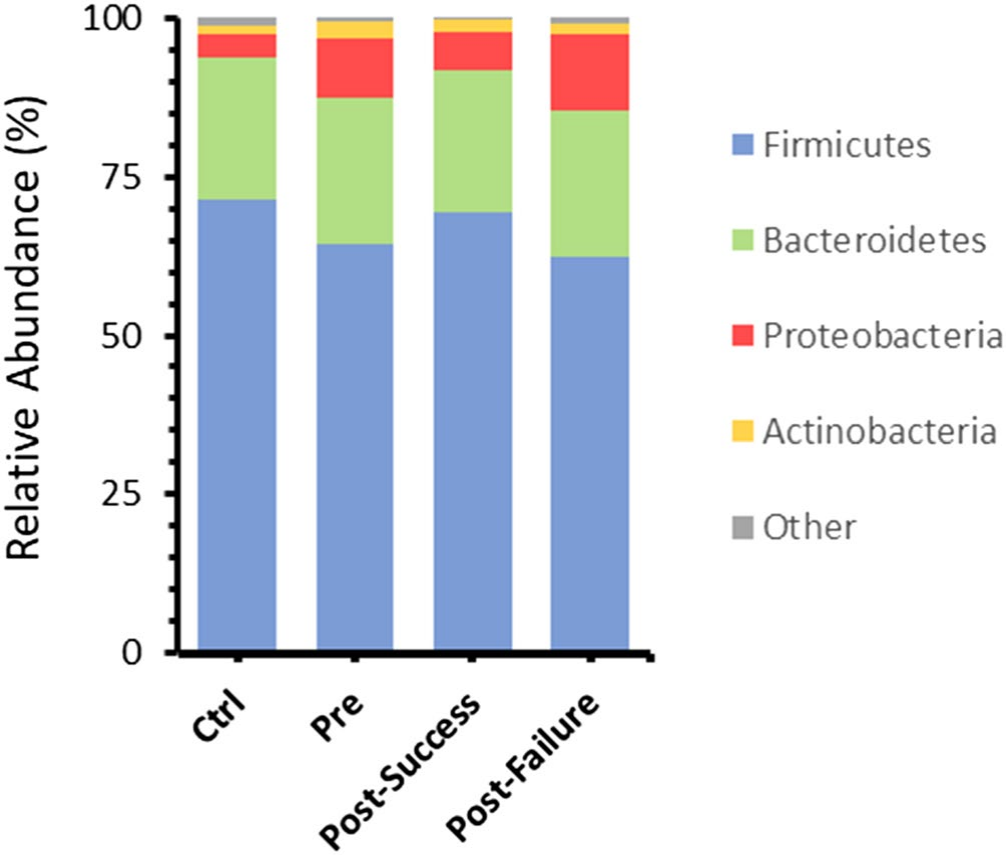
Bar charts showing mean values of ASV abundances at the phylum level in the four different groups studied: healthy individuals (control), Crohn's disease patients pre-treatment (t0) and patients post-treatment (t1; in turn, classified into responders and non-responders).**.** “Other” refers to all phyla with an initial representation less than 2% of abundaces, including Euryarchaeota, Actinobacteria, Cyanobacteria, Elusimicrobia, Epsilonbacteraeota, Fusobacteria, Lentisphaerae, Patescibacteria, Tenericutes and Verrucomicrobia.

In addition, an initial taxonomic analysis at the phylum level indicated a significant increase in Proteobacteria, Actinobacteria and Fusobacteria in CD patients compared to healthy individuals (*p-value* < *0.05*) (Fig. 2).

**Figure 3:**
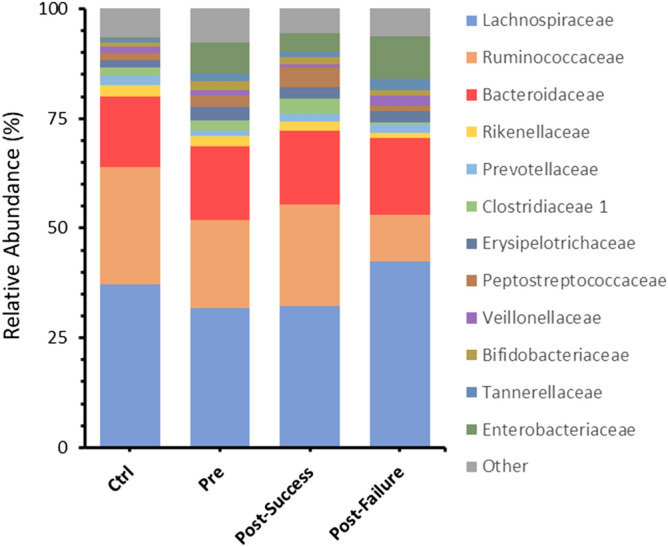
Bar charts showing mean values of ASV abundances at the family level in the four different groups studied: healthy individuals (control), Crohn's disease patients pre-treatment (t0) and patients post-treatment (t1; in turn, classified into responders and non-responders). “Other” refers to all families with an initial representation less than 2% of abundance.

Further taxonomic study at the family level indicated the pre-treatment group was significantly characterized by a lower representation of Ruminococcaceae and Christensenellaceae (*p-value* < *0.01*) and an increase in numerous bacterial families, notably Enterobacteriaceae, Erysipelotrichaceae and Bifidobacteriaceae (*p-value* < *0.05*) (Fig. 3).

**Figure 4:**
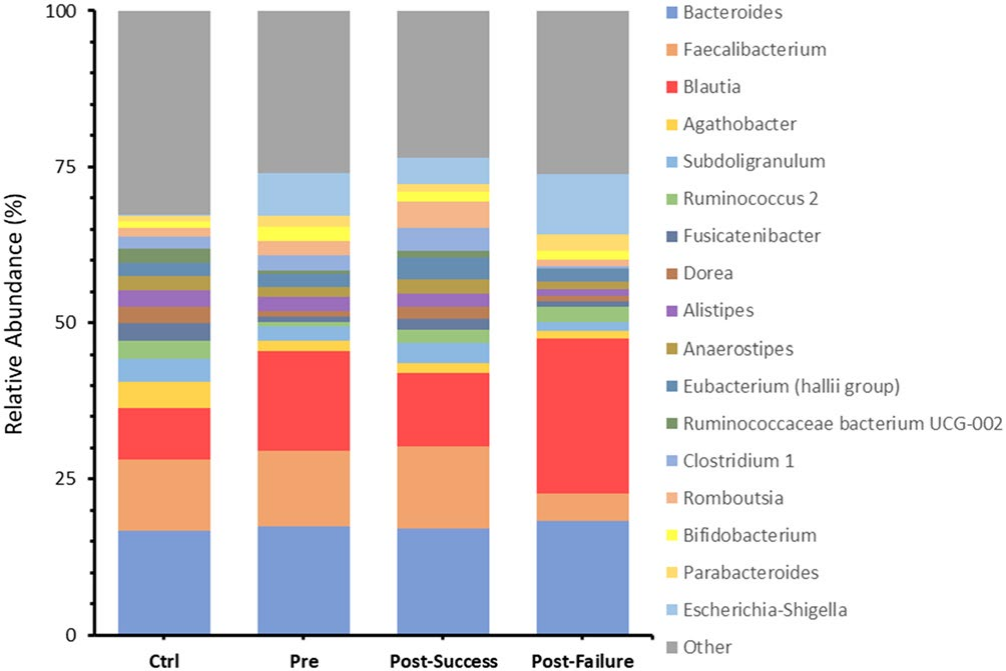
Bar charts showing mean values of ASV abundances at the genus level in the four different groups studied: healthy individuals (control), Crohn's disease patients pre-treatment (t0) and patients post-treatment (t1; in turn, classified into responders and non-responders). “Other” refers to all genera with an initial representation less than 2% of abundance.

At the genus level, the analyses carried out showed statistically significant differences in the concentration of a high number of bacterial genera. Notable among them was the decrease in *Ruminococcus, Agathobacter, Dorea* and *Fusicatenibacter* in CD, as well as the increase in the genus *Blautia, Escherichia/Shigella, Bifidobacterium* and *Lachnoclostridium* compared to the control group. Finally, at the species level, a lower representation of *Bacteroides plebeius, Alistipes obesi, Gabonia massiliensis* and *Faecalibacterium prausnitzii* was found in the CD microbiota compared to that of healthy controls (Fig. 4).

### Partial restoration of the intestinal microbiome in patients who responded to anti-TNF treatment

The included CD patients were classified as responders (n = 13) and non-responders (n = 14) after clinical and analytical evaluation 24 weeks after initiation of anti-TNF therapy, as described above. Biostatistical studies on alpha and beta diversity showed significant differences between the non-responder group and the control group, while no significant differences were observed between responders and the control group (Fig. 1A–C).

**Figure 5:**
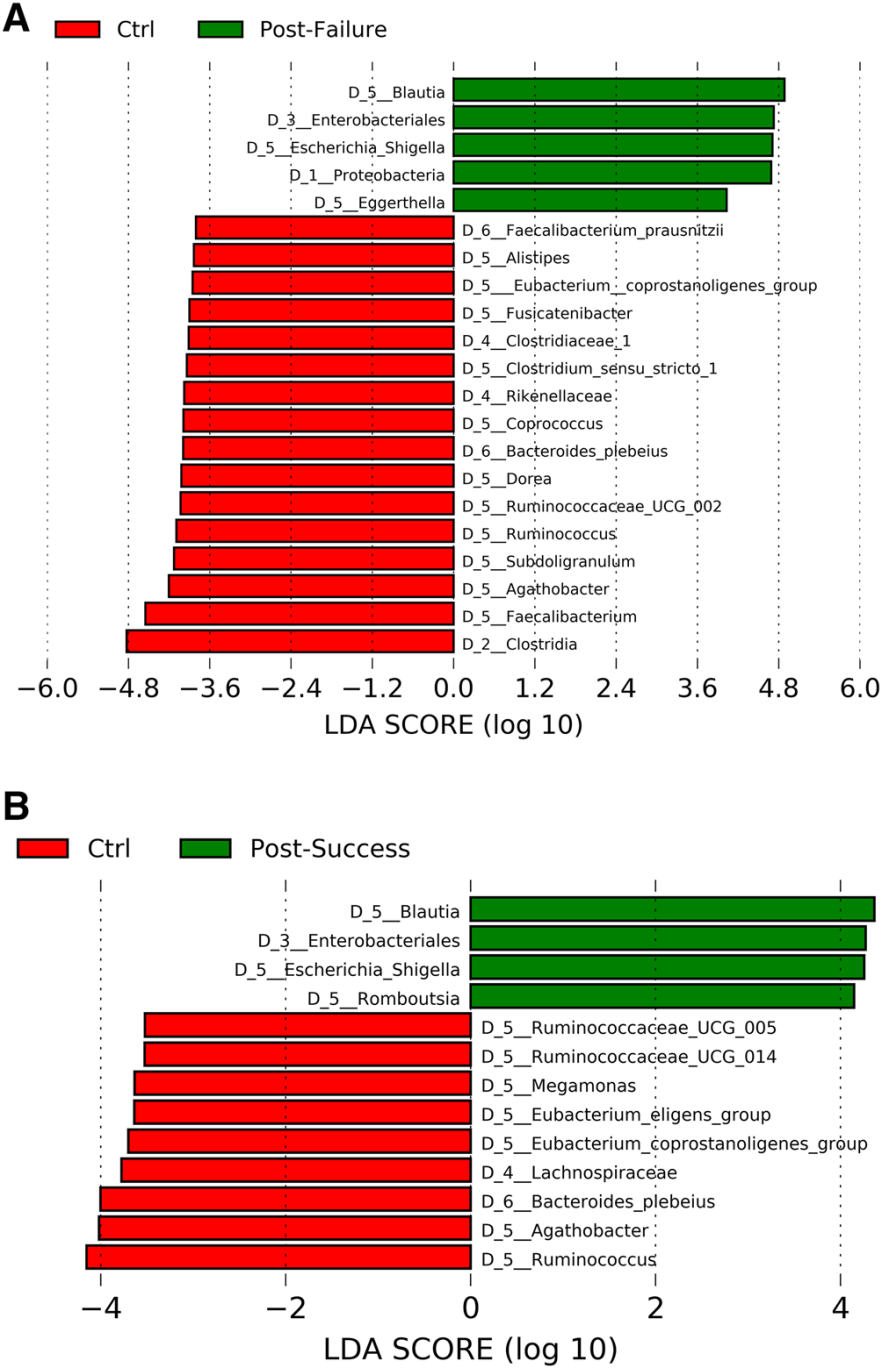
Differences in taxa abundance between (**A**) non-responders and (**B**) responders, compared to healthy individuals. The search for differential taxa was performed using LEfSe (Kruskal–Wallis test between classes, p-value < 0.05; threshold for discriminative taxa, logarithmic LDA score > 3.5).

In the taxonomic study, the concentration of genera such as *Escherichia/Shigella* increased significantly, and concentrations of *Faecalibacterium* and *Agathobacter* decreased in the non-responder group compared to healthy individuals. In the responder group, we highlight the importance of the elevated restoration in bacteria belonging to a determinant class such as Clostridia due to their anti-inflammatory metabolic functions; while those members of the order Enterobacterales, especially the genus *Escherichia/Shigella*, did not achieve the desired decrease in concentration but still showed significant differences between the microbiota of responders and that of the control group. Finally, the taxonomic level of species presented higher significant representations of *Bacteroides plebeius, Alistipes obesi and Faecalibacterium prausnitzii* in healthy individuals with respect to both groups of patients (Fig. 5A,B).

### Differences in genera in responders and non-responders

**Figure 6:**
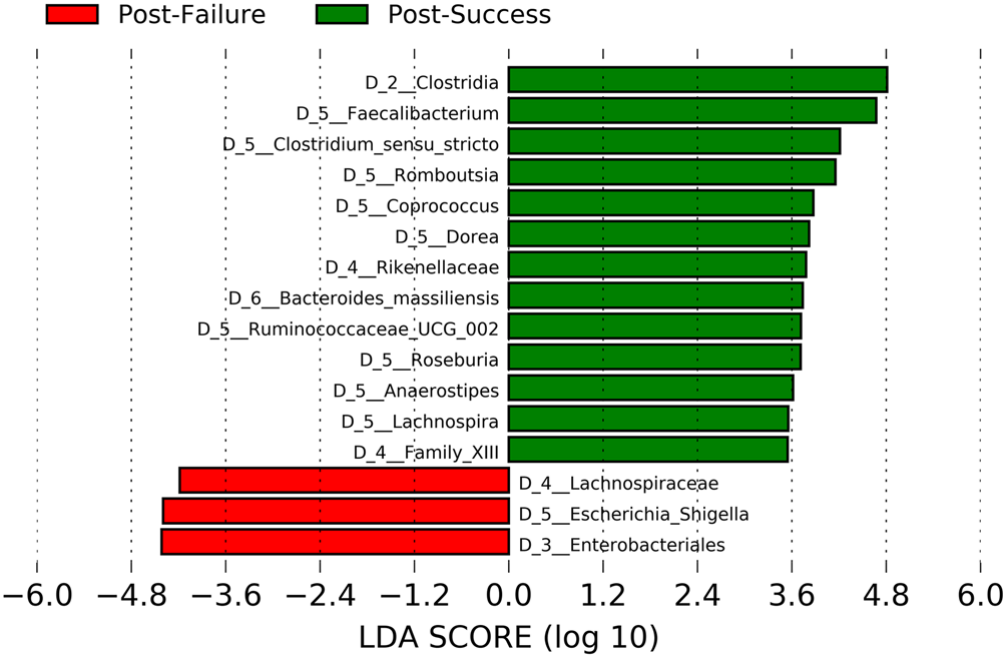
Differences in taxa abundance between responders and non-responders. Taxa with greater differences between the microbiota of both groups. The search for differential taxa was performed using LEfSe (Kruskal–Wallis test between classes, p-value < 0.05; threshold for discriminative taxa, logarithmic LDA score > 3.5).

Biostatistical studies on alpha and beta diversity indicated significant differences between the non-responders and the responders (Fig. 1A–C). The intestinal microbiome in patients who did not respond to anti-TNF was notable for the high concentration of bacteria belonging to the phylum Proteobacteria, specifically the order Enterobacteria and especially the genus *Escherichia/Shigella*. However, in the responder group, the phyla Bacteroidetes and Firmicutes had a greater significant representation; of the latter, those belonging to the class Clostridia predominated, particularly the genera *Faecalibacterium, Romboutsia, Coprococcus, Dorea, Roseburia, Anaerostipes* or *Lachnospira*, all of which had a log score above 3.5 (Fig. 6).

### The intestinal microbiome as a biomarker of therapeutic response through the *Faecalibacterium prausnitzii/Escherichia coli* (F/E) ratio

**Figure 7:**
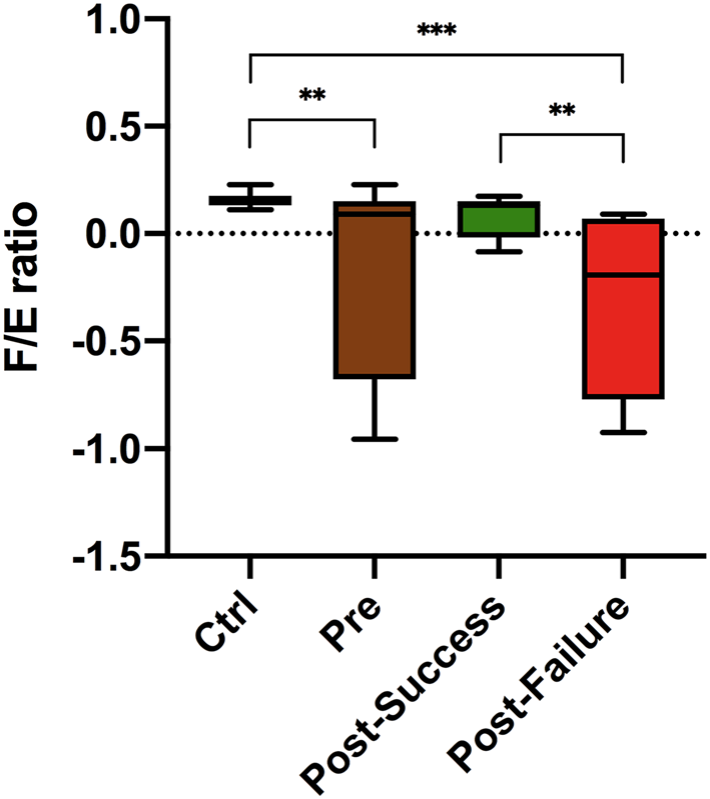
F/E ratio results. Statistical analysis using the Kruskal–Wallis test with Benjamini–Hochberg correction for multiple comparisons (false discovery rate). Asterisks refer to the corrected p-value, where * p-value < 0.05, ** p-value < 0.01 and *** p-value < 0.001.

The results obtained make it possible to choose two specific species, *Faecalibacterium prausnitzii* and *Escherichia coli*, as the main representatives of two genera strongly linked to intestinal eubiosis/dysbiosis in the context of inflammatory activity in CD and therefore the F/E ratio could be a marker of therapeutic response. The implementation of the ddPCR technique made it possible to evaluate the F/E ratio, finding statistically significant results between the control and pre-treatment groups, the controls and non-responders and, above all, between responders and non-responders to the anti-TNF treatment studied. In addition, it should be noted that there were no significant differences between the control group and anti-TNF responders (Fig. 7).

**Figure 8:**
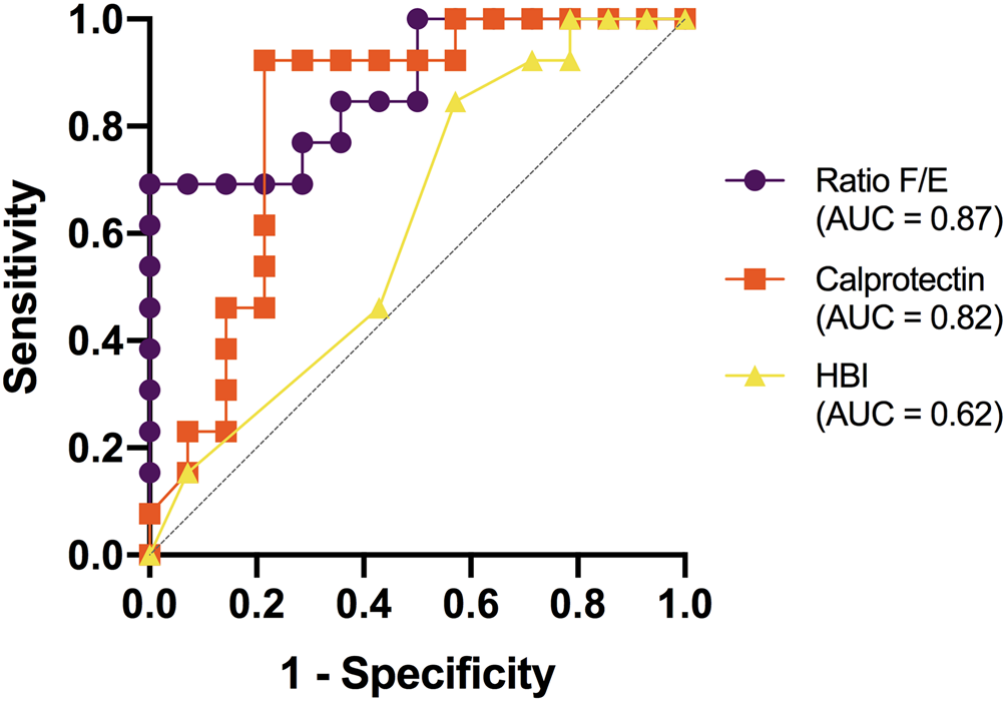
ROC curves for evaluation of the potential of the F/E ratio, calprotectin and HBI as biomarkers of therapeutic response. The value of the area under the curve (AUC) indicates the ability of each set of parameters studied as biomarkers of response in anti-TNF treatments.

Finally, an ROC (receiver operating characteristic) curve analysis was performed^23^, with an area under the curve (AUC) value for the F/E ratio equal to 0.874 (95% CI 0.741–1.000; p = 0.001), which is superior to other biomarkers currently used to monitor patients with CD in routine clinical practice, such as calprotectin fecal (FC) with AUC = 0.816 (95% CI 0.642–0.989; *p* = *0.005*), and Harvey-Bradshaw index with AUC = 0.615 (95% CI 0.400–0.831; *p* = *0.385*) (Fig. 8), or other possible indicators such as Microbial Dysbiosis Index (MD-index) with AUC = 0.692 (95% CI 0.349–0.836, *p* = *0.385*), log[*Escherichia coli*] with AUC = 0.602 (95% CI 0.383–0.820; *p* = *0.369*) and log[*Faecalibacterium prausnitzii*] with AUC = 0.824 (95% CI 0.668–0.981; *p* = *0.004*).

The cut-off points calculated using the Youden index of maximum sensitivity and specificity have been for the F/E ratio < 0.037 (Youden index = 0.495; sensitivity = 0.923 and specificity = 0.429) and for FC = 100.5 μg/g (Youden index = 0.709; sensitivity = 0.923 and specificity = 0.786).

## Materials and methods

### Patient cohorts and clinical evaluation

A total of 43 subjects were evaluated, divided into a cohort of 27 CD patients (15 men), with a mean age of 41.4 ± 17.4 years (Table 1) who required anti-TNF treatment to control the disease, and a group of 16 healthy individuals (8 men) with a mean age of 29.3 ± 7.2 years, family members of the CD patients, who shared the same lifestyle, eating habits and similar epidemiological characteristics, with no significant differences with respect to the main clinical and epidemiological variables analyzed.

**Table 1:** Clinical and demographic variables of the patients (total n = 27).

Variables	N (%); mean ± SD
Men	19 (55.9)
Age (years)	34.6 ± 15.0
**Extent of disease**	
Ileum	15 (44.1)
Ileocolic	14 (41.2)
Colon	4 (11.8)
Upper gastrointestinal tract	1 (2.9)
**Pattern of disease**	
Inflammatory	20 (58.8)
Stenosing	11 (32.4)
Fistulizing	3 (8.8)
Associated perianal disease	7 (20.6)
**Smoking habit**	
Smoker	17 (50)
Former smoker	2 (5.8)
Never smoked	15 (44.1)
Associated extra-intestinal manifestations	6 (17.6)
**Anti-TNF**	
IFX	19 (55.9)
ADA	15 (44.1)
**Anti-TNF indication**	
Failure of IS	7 (20.6)
Corticosteroid-dependent	5 (14.7)
Flare-up activity	11 (32.4)
Fistulizing disease	2 (5.9)
Top-down strategy	7 (20.6)
**Treatments before starting anti-TNF**	
5-ASA	19 (55.9)
Azathioprine	23 (67.6)
Corticosteroids	22 (64.7)
Methotrexate	2 (5.9)
**Concomitant treatment with anti-TNF**	
5-ASA	7 (20.6)
Azathioprine	19 (55.9)
Corticosteroids for induction	15 (44.1)
Methotrexate	1 (2.9)
**Baseline clinical status (Harvey-Bradshaw Index)**	
Moderate/severe activity (≥ 8 points)	17 (50)
Baseline endoscopy	23 (67.6)
Moderate/severe activity	20 (87)
Baseline MRI enterography	20 (58.8)
Moderate/severe activity	15 (75)

*IFX* infliximab, *ADA* adalimumab, *IS* immunosuppressive drugs, *5-ASA* 5-aminosalicylic acid, *MRI* magnetic resonance imaging.

The patients were recruited from eight hospitals in the Valencian Community. Treatment with anti-TNF was initiated under medical prescription according to routine clinical practice and is listed in the data sheet. Administration of this drug was therefore not promoted by this study. The clinical and demographic variables are given in Table 1.

Excluded from the study were all subjects who had taken antibiotics, probiotics and/or PPIs during the 4 weeks prior to study inclusion; patients with chronic HCV hepatitis and chronic HIV infection; indication for anti-TNF other than for control of their luminal disease (e.g. enteropathic arthropathy, perianal disease, prevention of recurrence, etc.); patients with previous ileum or colon surgery; and previous anti-TNF treatment in the 24 weeks prior to commencement of the study.

The study patients were prospectively monitored during the first 6 months of anti-TNF treatment. The presence of inflammatory activity was determined by calculation of the Harvey-Bradshaw index, biological activity by means of laboratory analytical data including complete blood count, albumin, C-reactive protein and fecal calprotectin (FC) prior to anti-TNF exposure and at 3 and 6 months after treatment, as well as stool cultures and parasites in stools to rule out concomitant infection, all during follow-up and whenever clinical changes were seen in the patient, in accordance with routine clinical practice. Thus, with these data, each patient was classified as either a responder or a non-responder.

### Stool sample collection

Fecal samples were collected for analysis of intestinal microbiota, prior to exposure to the anti-TNF drug and 6 months after initiation of treatment, and stored in the laboratory of each center at – 20 °C.

All the subjects were given a 3-day dietary intake questionnaire prior to stool collection. The same 3-day diet was followed before the 6-month stool collection, in order to minimize dietary bias.

### Sequencing and bioinformatics

DNA was obtained from 200 mg of stool using the QIAamp PowerFecal Pro kit (QIAGEN), through mechanical and enzymatic lysis. The V3 and V4 region of the 16S rRNA subunit^2^ was then amplified and sequencing was performed following the Library Illumina 15044223 B protocol and the MiSeq platform (ILLUMINA) 2 × 300 bp configuration (ADM-Lifesequencing S.L., Valencia). Sequencing data were processed following the Microbiome bioinformatics protocol with QIIME2 (Quantitative Insights Into Microbial Ecology) version 2019.4^18^. Raw sequences were analyzed using DADA2 (Divisive Amplicon Denoising Algorithm) and clustered into Amplicon Sequence Variants (ASVs)^19,20^, (via q2‐dada2). All amplicon sequence variants (ASVs) were aligned with mafft (Katoh et al. 2002) (via q2‐alignment) and used to construct a phylogeny with fasttree2 (Price et al. 2010) (via q2‐phylogeny). Alpha‐diversity metrics (observed features and Faith’s Phylogenetic Diversity (Faith 1992)), beta diversity metrics (weighted UniFrac (Lozupone et al. 2007), unweighted UniFrac (Lozupone et al. 2005), Jaccard distance, and Bray‐Curtis dissimilarity), and Principle Coordinate Analysis (PCoA) were estimated using q2‐diversity after samples were rarefied to the minimum feature count (value of 2311.6) observed across all of them during alpha and beta diversity analyses. Taxonomy was assigned to ASVs using the q2‐feature‐classifier (Bokulich et al. 2018a) classify‐sklearn naïve Bayes taxonomy classifier at different levels, against the SILVA 16 s rRNA gene reference database (v132, set NR99)^21^.

### Search for and evaluation of potential biomarkers

To identify potential non-invasive biomarkers from the characterization of the intestinal microbiome in various stages of CD, LEfSe (Linear Discriminant Analysis Effect Size) was used^22^. In addition, the absolute quantification of *Faecalibacterium prausnitzii, Escherichia coli*, and the total bacteria species present was studied using the QX200 Droplet Digital PCR system (ddPCR; Bio-Rad Laboratories), following the ddPCR Multiplex Supermix (BIORAD) kit protocol^5,23–26^. For this, the appropriate primers and probes were selected to perform a triplex PCR together with the optimization of their concentrations and thermal programs^5,23–26^. The QuantaSoft program version 1.7.4 was used to export the recorded amplitude data. The Faecalibacterium prausnitzii/Escherichia coli (F/E) ratio analysis was normalized using the following equation ([log10 (copies/µL *F. prausnitzii*) – log10 (copies/µL *E. coli*)] / [log10 (copies/µL ARNr 16S])^27^_._

### Statistical analysis

The study of ROC curves (AUC values)^28^, were carried out using the statistical program IBM SPSS Statistics 26.0. The statistical analysis to obtain the concentration values required for the calculation of the F/E ratio through the ddPCR technique was performed through an adaptation of ddPCRmulti, a semi-automatic application based on R version 3.6^23^. In the ROC curves of each of the possible indicators associated with the success of treatment with anti-TNFs, the cut-off values for greater sensitivity and specificity have been calculated using the index Youden (sensitivity + specificity-1). In the bivariate analysis, the Bonferroni correction for multiple comparisons was made, setting the level of significance at *p* < *0.0125*.

### Ethical considerations

The study was carried out in strict accordance with the international ethical recommendations for research and clinical trials in humans contained in the Declaration of Helsinki and was classified by the Spanish Agency of Medicines and Healthcare Products as a prospective follow-up post-authorization study on February 12, 2015 (FXC-TNF-2015-01). It was subsequently approved by the Clinical Research Ethics Committee of Sagunto Hospital on July 22, 2015 (FXC-TNF-2015–01) and endorsed by the local ethics committees of all the participating centers. Written informed consent was obtained from the patients and the study participants.

## Discussion

This study, in agreement with others, shows that CD is characterized by dysbiosis, i.e. an imbalance in gut bacteria compared to healthy persons, in terms of low alpha and beta biodiversity^29–32^, and by a marked reduction in bacteria belonging to the class Clostridia, mainly SCFA-producing genera, with anti-inflammatory properties. In contrast, there was an increase in the representation of phylum Proteobacteria, mainly of the genus *Escherichia/Shigella* (pro-inflammatory). However, a higher relative abundance of the genus *Blautia* was also found in patients, while other studies reported its decrease in CD^33,34^.

Regarding the changes observed after anti-TNF treatment, a therapy that has demonstrated a high remission rate in CD patients, we saw that responders presented a partial restoration of the microbiome characteristic of healthy individuals, that is, they presented a tendency towards eubiosis, with a significant increase in bacteria belonging to the class Clostridia, with anti-inflammatory metabolic functions. However, those members of the order Enterobacterales, especially the genus *Escherichia/Shigella* (pro-inflammatory), although significantly reduced in the responders compared to baseline, did not reach significance compared to healthy controls. Accordingly, this is considered a partial restoration^14,35^.

The study of differential microbiota in anti-TNF responders and non-responders enabled us to establish two bacterial species, *Faecalibacterium prausnitzii* and *Escherichia coli*, as those with the most suitable characteristics for use as biomarkers of therapeutic response^3–5,11,12^, given the high relative abundance of the genus *Escherichia/Shigella* in non-responders and a greater representation of the genus *Faecalibacterium* in responders to this therapy. With all this, we calculated the F/E ratio, previously described in another study, as a marker of dysbiosis^14^, through its absolute quantification by ddPCR and found significant differences between responders and non-responders. In contrast, no significant differences were found between responders and healthy controls. This is therefore a promising result in the search for rapid methods of analysis, such as biomarkers of therapeutic response.

The results obtained reinforce the importance of the role of the intestinal microbiome, particularly the loss of capacity in CD patients for inflammation regulation and epithelial repair, highlighting its partial recovery in responding patients after an increase in the abundance of certain SCFA-producing genera such as *Faecalibacterium*. These fatty acids, produced by the bacterial fermentation of non-digestible carbohydrates, constitute a source of colonic energy, in addition to their influence on the determination of the intestinal environment, influencing transit, nutrient uptake, pH and microbial balance. Thus, they also present important immunomodulatory and anti-inflammatory properties, mainly through the control of T-regulatory cell homeostasis. For example, the secretion of metabolites from *Faecalibacterium prausnitzii* can block the activation of NF-kB and the production of IL-8, affecting the inflammatory cascade^5,36,37^.

The statistical analyses performed show promising results concerning the potential of the F/E ratio as a biomarker of therapeutic response based on the relationship between the intestinal microbiome and CD. Indeed, it was superior to other biomarkers such as FC and the Harvey-Bradshaw index currently used in monitoring this disease in routine clinical practice. The use of the F/E ratio, either alone or in combination with other biomarkers, could be a new therapeutic objective in this disease, although validation studies are needed prior to its introduction into clinical practice. New clinical studies may provide further evidence of its potential use as a biomarker with non-invasive anti-TNF response samples in CD, allowing decision-making about therapy to manage CD, modifying treatments according to whether or not it is possible to achieve a microbiota similar to that of healthy subjects. In this way, the course of the disease can be changed based on its pathogenesis, i.e. dysbiosis, and not, as has been done up to now, through its consequences such as the inflammatory cascade using measurements such as FC or invasive techniques such as endoscopy.

Thus, with the cut-off points obtained, the number of anti-TNF responders coincides with both parameters (fecal calprotectin and F/E index), whose distribution is presented in Table 2.

**Table 2:** True positives, false positives, sensitivity, specificity and predictive values obtained in the sample analyzed using the F/E index and FC.

	F/E index ≥ 0.037 n (%)	FC ≤ 100.5 μg/g N (%)
Anti-TNF responders (n = 13)	12 (92.3)	12 (92.3)
Anti-TNF non-responders (n = 14)	6 (42.9)	3 (21.4)
P value test Chi square	0.006	< 0.001
Sensitivity	92.3%	92.3%
Specificity	57.1%	78.6%
Positive predictive value	66.7%	80.0%
Negative predictive value	88.9%	91.7%

Although this cut-off point does not statistically exceed fecal calprotectin, it could be a good predictor of restoration of intestinal microbiota associated with a response to anti-TNF treatment. It could even predict response by determining it before the start of treatment if these values of sensitivity and specificity ​​were maintained when expanding the sample size in the future. In this way, establish which patients to offer anti-TNF and in whom we should look for other alternatives.

Among the strengths, in addition to the implications for clinical practice by establishing the F/E ratio as a novel non-invasive tool in therapeutic decision-making, it should be noted that the healthy individuals included were family members of the patients, with absolutely similar diets and therefore perfectly comparable. Moreover, to further minimize dietary bias, at six months the patients consumed the same diet for the 3 days prior to the first stool collection. This was not done in any of the other studies published to date.

One of the limitations is that as this was an observational, non-interventional study, confounding factors may have been present between treatment allocation and outcomes, in comparative analyses of response rates. In addition, patient samples that do not represent real-world clinical practices may have been included, for example, heterogeneous patients with respect to their previous anti-TNF treatments. Another limitation is the reduced number of patients included due to the inherent cost of the technique, which prevented the inclusion of the same number of healthy subjects as patients. Furthermore, although it is a multicenter study, the number of patients who are incorporated into anti-TNF treatment and who meet all the inclusion and exclusion criteria is limited, even more so the number of blood relatives living with the patient and with a diet similar to that used as a control. It should be noted, however, that our sample size for the CD patient group was similar to that published to date in studies with a similar design^38^ and that the results allow this number of patients to respond to the objectives set.

## Conclusions

CD is characterized by dysbiosis, with reduced class Clostridia bacteria, mainly genera associated with the SCFA production. Anti-TNF therapy in responding patients partially restores the intestinal microbiome to become almost similar to the microbiota of healthy individuals. The F/E ratio, as a non-invasive biomarker, allows differentiation between responders and non-responders, with good levels of sensitivity and specificity compared to other biomarkers currently used in clinical practice.
